# Sink survey to investigate multidrug resistance pattern of common foodborne bacteria from wholesale chicken markets in Dhaka city of Bangladesh

**DOI:** 10.1038/s41598-022-14883-7

**Published:** 2022-06-25

**Authors:** Mst. Sonia Parvin, Md. Yamin Ali, Amit Kumar Mandal, Sudipta Talukder, Md. Taohidul Islam

**Affiliations:** 1 Population Medicine and AMR Laboratory, Department of Medicine, Faculty of Veterinary Science, Bangladesh Agricultural University, Mymensingh, 2202 Bangladesh; 2 Department of Livestock Services, Farmgate, Dhaka, 1215 Bangladesh

**Keywords:** Antimicrobial resistance, Water microbiology

## Abstract

Antimicrobial resistance (AMR) among foodborne bacteria is a well-known public health problem. A sink survey was conducted to determine the AMR pattern of common foodborne bacteria in cloacal swab of broiler chickens and sewage samples from five wholesale chicken markets of Dhaka city in Bangladesh. Bacteria were identified by culture-based and molecular methods, and subjected to antimicrobial susceptibility testing. Resistance genes were identified by multiplex PCR and sequencing. Multidrug resistance (MDR) was observed in 93.2% of *E. coli*, 100% of *Salmonella* spp., and 97.2% of *S. aureus* from cloacal swab samples. For sewage samples, 80% of *E. coli*, and 100% of *Salmonella* and *S. aureus* showed MDR. Noteworthy, 8.3% of *S. aureus* from cloacal swab samples showed possible extensively drug resistance. Antimicrobial resistance genes (beta-lactamase—*bla*TEM, *bla*SHV; quinolone resistance gene—*qnr*S) were detected in a number of *E. coli* and *Salmonella* isolates from cloacal swab and sewage samples. The methicillin resistance gene (*mec*A) was detected in 47.2% and 25% *S. aureus* from cloacal swab and sewage samples, respectively. The findings envisage the potential public health risk and environmental health hazard through spillover of common foodborne MDR bacteria.

## Introduction

Foodborne pathogens cause significant illnesses, death and expenses in the world^1,2^. The foodborne bacteria are accountable to establish a significant burden of infection in developing and developed countries but the impact is very high in developing countries like Bangladesh^3,4^. Furthermore, due to the growing development and spread of antimicrobial resistance (AMR) among foodborne bacteria, the burden of foodborne infections representing a significant and pervasive threat to the general wellbeing and economy^1,5,6^. Normally, foodborne bacteria exists in the gastrointestinal tract of chickens, and presently, chickens are viewed as the essential driver for various foodborne bacterial infections^1,7,8^. Foodborne bacteria are transmitted from chickens to individuals through exposure to chickens, the ingestion of bacteria carrying resistance, direct contact, and amplification and passage of resistant strains into the environment^7^. Thus, antibiotic resistant bacteria exist across the poultry, human, and environment, and there is interlinked sharing of these bacteria in this triad^7,9,10^.

The presence of extended-spectrum β-lactamases (ESBL) in *E. coli* and *Salmonella* spp. have exacerbated the worldwide circumstance of AMR in view of their capacity to hydrolyze and inactivate β-lactam antibiotics, including third and fourth generation cephalosporins, which are generally used to treat serious infections caused by members of the *Enterobacteriaceae* family^11,12^. ESBLs are generally situated on versatile genetic elements, for example, plasmids or integrons, which can encourage their portability from bacterial species to others by horizontal gene transfer^13^. ESBL-producing bacteria have also shown co-resistance to quinolones, aminoglycosides, and sulphonamides, contributing to the emergence of MDR^14^. In the past few decades, methicillin resistant *S. aureus* (MRSA) has emerged, which poses a serious public health threat in both communities and hospitals, and poultry since the treatment of infections are more difficult when encountering resistance^15,16^. According to WHO, MRSA has been listed as one of the high-priority antibiotic-resistant pathogens^17^. Along with antimicrobial-resistant bacteria, the presence of antimicrobial resistance genes (ARGs) in chickens and environments is a problem due to the possibility of gene transfer from bacteria harboring resistance genes to susceptible bacteria^7^. ARGs such as *bla*TEM and *bla*SHV (resistance to broad spectrum β-lactams), *bla*CTX-M-1 and *bla*CTX-M-2 (resistance to extended spectrum β-lactams), *qnrA*, *qnrB* and *qnrS* (reduced susceptibility to fluoroquinolones), and *mecA* (resistance to methicillin) have been reported worldwide including Bangladesh^9,10,18–22^. In Bangladesh, *bla*TEM (41–91.3%), *bla*SHV (85%), *bla*CTX-M-1 (28–94.4%), *qnrB* (9%), and *qnrS* (27.7–72.2%) have been detected in *E. coli* isolated from droppings of chickens and supply water^9,18,23–26^. In addition, the prevalence of *mecA* gene (19.2%) in *S. aureus* in Bangladesh has been reported in cloacal swab^21^. Therefore, it is essential to perform research to provide various scientific information for clinicians to select proper antibiotics to treat common foodborne bacterial infections, and also for public health authorities to make up regulatory standards and guidelines to control dissemination of ARGs.

In Bangladesh, wholesale markets are the important sources of live poultry supply to consumers, which are particularly located in larger cities, where wholesalers sell their poultry to retailers, hawkers and even directly to restaurants^27^. Different species of poultry are aggregated in wholesale markets, where multiple poultry species are housed together for sale that can facilitate spread of bacterial and viral infections among poultry and from poultry to humans. Furthermore, wholesale markets have been rendered as the potential reservoirs of poultry associated foodborne antimicrobial resistant bacteria and ARGs^28^. Several research works have been conducted regarding the prevalence, AMR pattern of *E. coli* and *Salmonella* spp. from cloacal swab samples of broiler chickens sold in live bird markets in Bangladesh^18,23,26,29^. However, the occurrence of AMR foodborne bacteria in poultry sold in wholesale market has not been examined. The present study was, therefore, conducted to determine the AMR pattern of common foodborne bacteria isolated from cloacal swab and sewage samples from wholesale chicken markets of Dhaka city in Bangladesh. We also focused on the determination of antimicrobial resistance genes in common foodborne bacteria.

## Results

### Demographic information and disposal practices of wholesale chicken markets

We observed that in all five wholesale chicken markets, poultry, especially chickens were mainly brought from different districts (second tier of regional administrative area) of Bangladesh. Approximately 5000–10,000 chickens were sold each day per wholesale chicken market except Mohammadpur Krishi Market where more than 10,000 chickens vended each day. During sampling, we observed that different types of poultry such as broiler, layer, cockerel, backyard chickens, turkey, duck, and pigeon were kept together in a very compact stall throughout the day for selling. These poultry were mainly distributed to various retail markets, supershops, hotel, restaurant, and community center throughout Dhaka city (Supplementary Table S1).

Liquid wastes such as blood, waste water mixed with droppings were washed out and drained into nearby Buriganga River, which flows past the southwest outskirts of the capital city Dhaka. Solid wastes such as poultry plumes, offales and leftover feed were disposed into drum, which was then collected by the waste collectors and disposed into the municipal corporation dustbin situated close to the road. However, in Karwan Bazar Kitchen Market, solid wastes were normally kept into the nearby bucket, which were offered to different merchants who purchased and sold these to fish ranchers.

### Distribution of *E. coli*, *Salmonella* spp. and *S. aureus*

**Figure 1:**
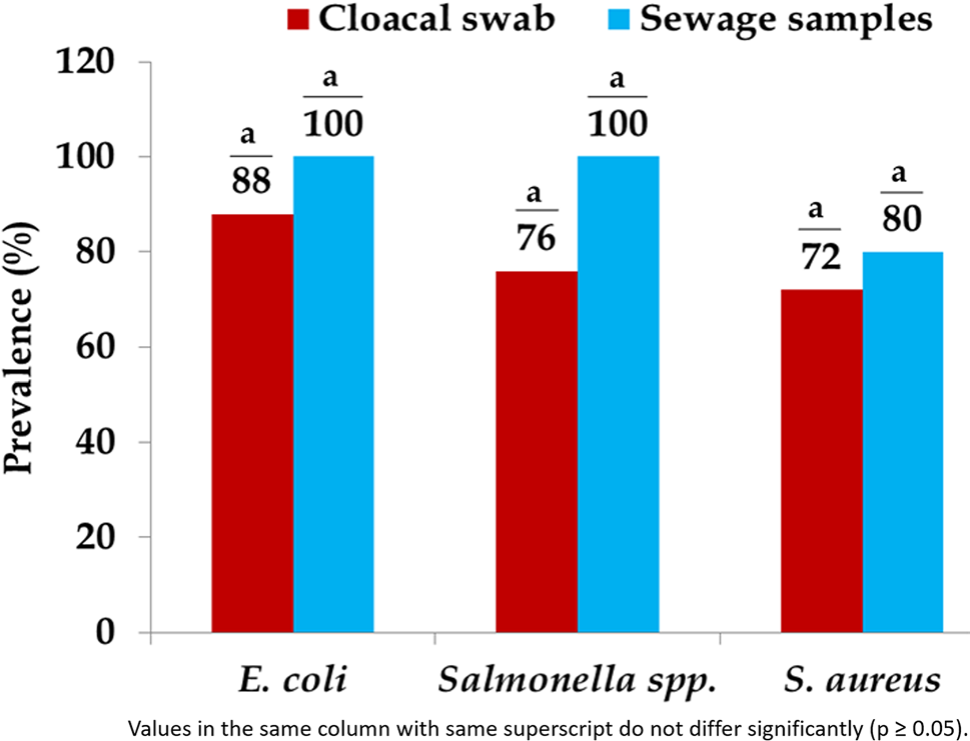
Prevalence of *E. coli*, *Salmonella* spp. and *S. aureus* isolated from cloacal swab and sewage samples following enrichment.

Of the 55 samples (50 pooled cloacal swab and 5 pooled sewage), the *E. coli* were detected in 88% (44/50) of cloacal swab and 100% (5/5) of sewage samples while 76% (38/50) of cloacal swab and 100% (5/5) of sewage samples were positive for *Salmonella* spp. The occurrence of *S. aureus* was 72% (36/50) and 80% (4/5) in cloacal swab and sewage samples, respectively (Fig. 1). There was no significant difference among three types of bacteria isolated from both samples. All isolates of *E. coli*, *Salmonella* spp., and *S. aureus* produced expected product size by PCR.

The market-wise analysis revealed that, the occurrence of *E. coli* isolated from cloacal swab samples was significantly higher in Karwan Bazar Kitchen Market and Mohakhali Kacha Bazar (100%) compared with Mirpur-1 Kacha Bazar (60%). For *Salmonella* spp., significantly higher occurrence was observed in Gulistan Kaptan Bazar (90%) than Mohakhali Kacha Bazar (60%). In case of *S. aureus*, all the markets had high occurrence (70–80%). For sewage samples, no significant differences were observed in the occurrence of *E. coli*, *Salmonella* spp. and *S. aureus* among five wholesale chicken markets (Supplementary Table S2). All the sewage samples were positive for *E. coli* and *Salmonella* spp. from five wholesale chicken markets. However, none of the isolates of *S. aureus* were recovered from Mohakhali Kacha Bazar.

**Figure 2:**
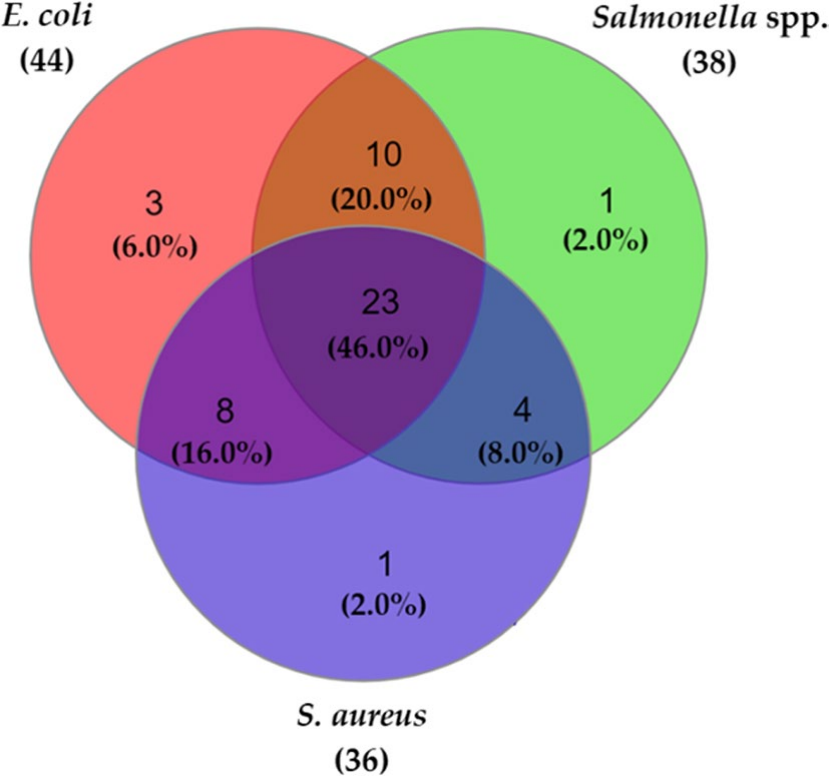
Venn-diagram showing co-isolation of *E. coli*, *Salmonella* spp. and *S. aureus* in cloacal swab.

Venn diagram displaying more than one bacteria (90%, 45/50) was co-isolated from cloacal swab samples from wholesale chicken markets. The occurrence of co-isolation with *E. coli*, *Salmonella* spp., and *S. aureus* was found highest (46%, n = 23), followed by *E. coli* and *Salmonella* spp. Single isolation with either *E. coli*, *Salmonella* spp. and *S. aureus* was found in 5 (10%) of cloacal swab samples (Fig. 2).

### Prevalence of ESBL-producing and methicillin resistant bacteria

**Figure 3:**
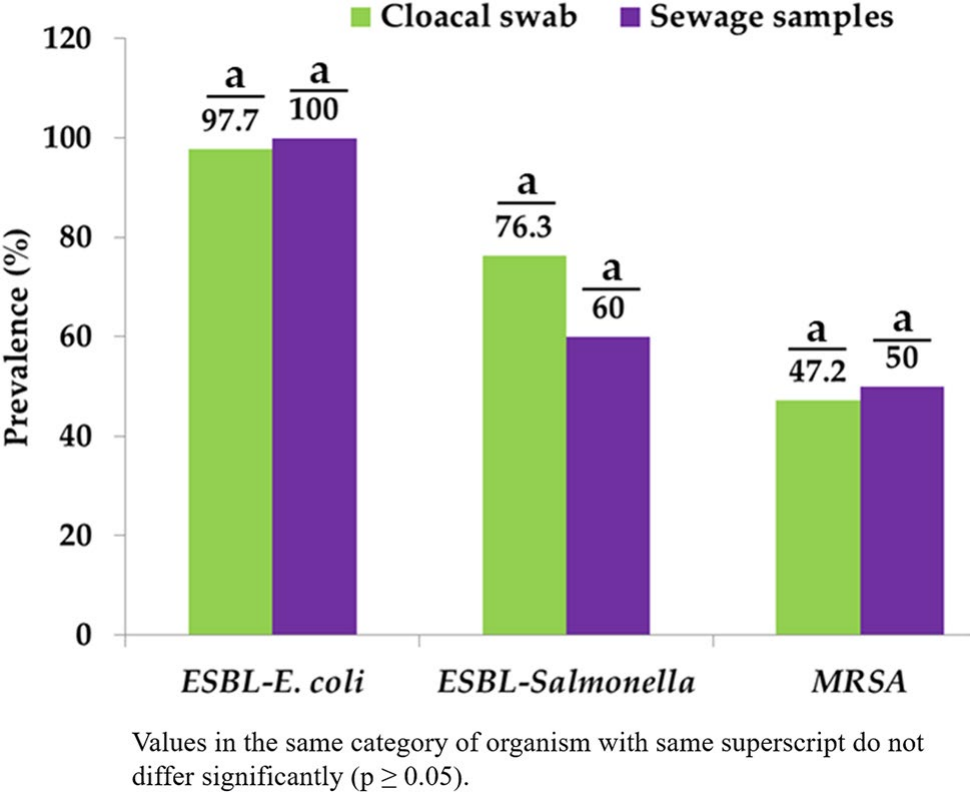
Prevalence of ESBL-producing *E. coli* and *Salmonella* spp., and methicillin-resistant *S. aureus* (MRSA) isolated from cloacal swab and sewage samples.

The prevalence of ESBL-producing *E. coli* from cloacal swab and sewage samples were 97.7% (43/44) and 100% (5/5), respectively, while the prevalence of ESBL-producing *Salmonella* spp. was 76.3% (29/38) in cloacal swab and 60% (3/5) in sewage samples. Moreover, methicillin resistant *S. aureus* was found in 47.2% (17/36) and 50% (2/4) of cloacal swab and sewage samples, respectively (Fig. 3).

### Distribution of MDR *E. coli*, *Salmonella* spp., and *S. aureus*

Noteworthy, in this study, we observed that 3 isolates (8.3%) of *S. aureus* from cloacal swab samples showed possible extensively drug resistance (pXDR), which was resistant to 9 or 10 out of 11 antimicrobial classes. The prevalence of MDR *E. coli*, *Salmonella* spp., and *S. aureus* recovered from cloacal swab samples were 93.2% (41/44), 100% (38/38) and 97.2% (35/36), respectively. Furthermore, half of the isolates of *E. coli* and *Salmonella* spp., and 44.4% isolates of *S. aureus*, resistant to 6–8 antimicrobial classes were observed higher compared with 3–5 and ≥ 9 classes of antimicrobials. For sewage samples from wholesale chicken markets, it was observed that all the isolates of *Salmonella* spp. and *S. aureus*, and 80% isolates of *E. coli* from sewage samples were MDR. The majority of *E. coli*, *Salmonella* spp. and *S. aureus* isolates were mostly resistant to 6–8 antimicrobial classes. One-fourth (20%) of the *Salmonella* spp. isolates showed resistance to ≥ 9 classes. Significant differences were observed among the three types of bacteria against each category of antimicrobial classes in both cloacal swab and sewage samples (Table 1).

**Table 1:** Multidrug resistance patterns observed among *E. coli*, *Salmonella* spp., and *S. aureus* isolated from cloacal swab and sewage samples.

Antimicrobial class	No. (%) of isolates					
	Cloacal swab			Sewage samples		
	*E. coli* (n = 44)	*Salmonella* spp. (n = 38)	*S. aureus* (n = 36)	*E. coli* (n = 5)	*Salmonella* spp. (n = 5)	*S. aureus* (n = 4)
3–5	14 (31.8)^ab^	10 (26.3)^a^	16 (44.4)^b^	1 (20)^a^	1 (20)^a^	2 (50)^b^
6–8	22 (50)^a^	19 (50)^a^	16 (44.4)^a^	3 (60)^a^	3 (60)^a^	2 (50)^a^
≥ 9	5 (11.4)^a^	9 (23.7)^b^	3 (8.3)^a^	0	1 (20)	0
Total	41 (93.2)	38 (100)	35 (97.2)	4 (80)	5 (100)	4 (100)

^a,b^Values with different letters in the same row of antimicrobial class for each sample type differ significantly (p ≤ 0.05).

Market-wise distributions revealed that all the markets had high occurrence of MDR *E. coli* (77.8–100%), *Salmonella* spp. (100%), and *S. aureus* (85.7–100%) in cloacal swab samples. In case of sewage samples, cent percentages of MDR *E. coli*, *Salmonella* spp., and *S. aureus* were also observed in all the markets (Supplementary Table S3).

**Figure 4:**
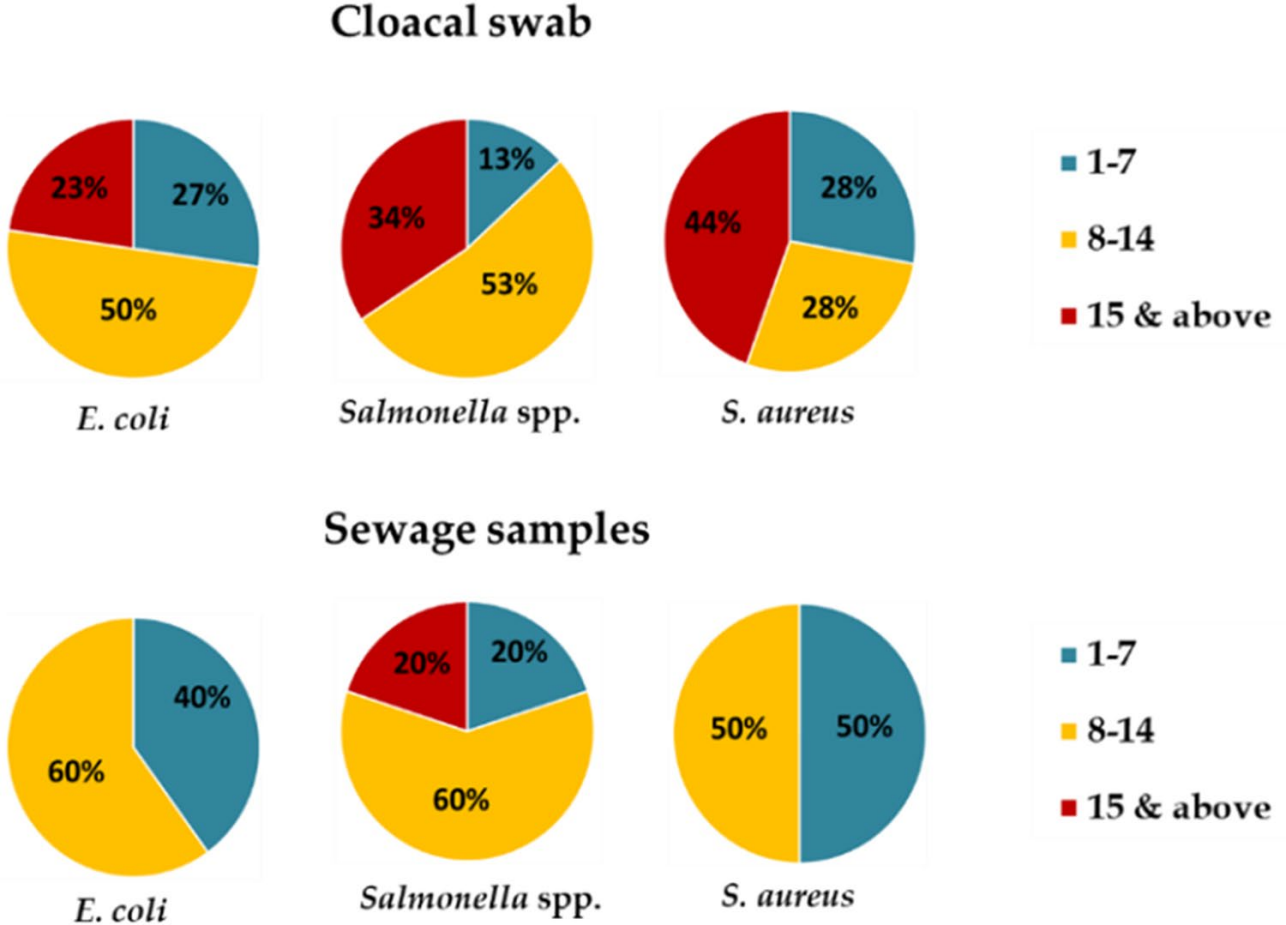
Resistance distribution of *E. coli*, *Salmonella* spp., and *S. aureus* to number of antimicrobial agents in cloacal swab and sewage samples.

The antimicrobial susceptibility testing revealed that all isolates of *E. coli*, *Salmonella* spp. and *S. aureus* recovered from cloacal swab and sewage samples were resistant to at least one, and up to fifteen or more antimicrobial agents (Fig. 4). The highest percentage of *E. coli* (50%) and *Salmonella* spp. (53%) isolates recovered from cloacal swab were resistant to 8–14 antimicrobial agents. On the other hand, 28% isolates of *S. aureus* were resistant to 8–14 antimicrobials. Resistance to 1–7 antimicrobials were observed in 27%, 13%, and 28% isolates of *E. coli*, *Salmonella* spp., and *S. aureus*, respectively. Of note, a certain percentages of *E. coli* (23%), *Salmonella* spp. (34%), and *S. aureus* (44%) isolates displayed resistance to 15 or more antimicrobials. In case of sewage samples from wholesale chicken markets, more than 50% isolates of *E. coli*, *Salmonella* spp. and *S. aureus* were resistant to 8–14 antimicrobial agents. A few percentage (20%) of *Salmonella* spp. isolates showed resistance to 15 or more antimicrobials.

An UpSet analysis of AMR revealed that the highest single resistance in *E. coli* isolated from cloacal swab was most frequently observed to pefloxacin (88.6%, n = 39), ampicillin (86.4%, n = 38), trimethoprim-sulfamethoxazole (81.8%, n = 36), amoxicillin-clavulanic acid (75%, n = 33), nalidixic acid (70.5%, n = 31) and, ofloxacin and doxycycline (63.6%, n = 28), whereas resistance to tigecycline, polymyxin B, piperacillin–tazobactam, and cefepime were observed to be lowest (2.3%, n = 1). Interestingly, none of the isolates of *E. coli* from cloacal swab was resistant to cephalexin, cefuroxime, cefoxitin and aztreonam. Forty three different antimicrobial resistance patterns were detected among *E. coli* isolates (Supplementary Fig. S1). The most common pattern was PEF-AM-SXT-AMC-NA-OFX-DO-CIP-GAT-IPM-MEM-NX-LEV-CAZ-C (n = 2). Notably, around 60% isolates of *E. coli* resisted carbapenems mainly imipenem and meropenem.

Furthermore, *Salmonella* spp. from cloacal swab expressed the highest resistance to polymyxin B and doxycycline (100%, n = 38), followed by nalidixic acid and colistin (97.4%, n = 37), pefloxacin and trimethoprim-sulfamethoxazole (84.2%, n = 32), ampicillin (73.7%, n = 28), ciprofloxacin (68.4%, n = 26) and gatifloxacin (63.2%, n = 24). On the other hand, resistance to tigecycline, piperacillin–tazobactam and cephalexin were found lowest (2.6%, n = 1). *Salmonella* spp. isolates from cloacal swab didn’t show resistance against cefoxitin. Among 38 tested isolates, 37 different patterns were observed, in which the most common pattern was PB-DO-NA-CT-PEF-SXT-AM-CIP-GAT-OFX-CAZ (n = 2) (Supplementary Fig. S2). Another important finding of this study is that, 44.7% of *Salmonella* spp. isolates exhibits resistance to imipenem.

On the other hand, *S. aureus* from cloacal swab samples exhibited the highest resistance to nalidixic acid (94.4%, n = 34), followed by oxacillin (88.9%, n = 32), gatifloxacin (83.3%, n = 30), cefixime and cloxacillin (80.6%, n = 29), and doxycycline (72.2%, n = 26). However, 5.6% and 8.3% isolates of *S. aureus* showed lowest resistance to imipenem and vancomycin, respectively. The isolates showed 35 different antimicrobial resistance patterns, and the most frequent pattern was NA-OX-GAT-CFM-CX-DO-NX-OFX-CT-CAZ (n = 2) (Supplementary Fig. S3). Of note, more than half of the *S. aureus* isolates were found to be resistant to colistin, while none of the isolates were observed resistant to meropenem.

For sewage samples, *E. coli* isolates showed high resistance to pefloxacin and trimethoprim-sulfamethoxazole (100%, n = 5), followed by ampicillin (80%, n = 4) and, imipenem, meropenem, nalidixic acid and doxycycline (60%, n = 3). Likewise, all isolates of *Salmonella* spp. showed resistance to nalidixic acid, pefloxacin, colistin, polymyxin B and doxycycline (100%). Resistance to ampicillin and trimethoprim-sulfamethoxazole (80%, n = 4), and gentamicin (60%, n = 3) were also observed very high. Notably, 40% of *Salmonella* spp. isolates exhibits resistance to imipenem. For *S. aureus*, resistance to ceftazidime (100%, n = 4) followed by nalidixic acid, ofloxacin, cefixime, cloxacillin and doxycycline (75%, n = 3) was observed high (Supplementary Table S4). Of note, half of the isolates were resistant to colistin.

### Prevalence of β-lactamase-, and PMQR-encoding genes in *E. coli* and *Salmonella* spp.

According to BSBL-encoding genes, all isolates of *E. coli* and *Salmonella* spp. from cloacal swab and sewage samples were positive for *bla*TEM gene. The *bla*SHV gene was detected in 20% (1/5) of *E. coli* isolates from sewage samples. None of the tested *E. coli* and *Salmonella* spp. isolates had ESBL-encoding gene in both types of samples. For PMQR genes, the *qnrA* gene was present in 9.1% (4/44) of the *E. coli* isolates from cloacal swab. The *qnrS* gene was simultaneously detected in 70.5% (31/44) and 80% (1/5) of *E. coli* isolates from cloacal swab and sewage samples, respectively. On the contrary, only one isolate (2.6%) of *Salmonella* spp. from cloacal swab samples contained the *qnrS* gene. The *qnrB* gene was completely absent in all isolates of *E. coli* and *Salmonella* spp. from both types of sample (Table 2). The partial sequencing of the eight *bl*aTEM, one *bla*SHV, two *qnrA* and seven *qnrS* genes (MT820235; MT820236; MT820237; MT820238; MT820247; MT820248; MT820301; MT820302; MT820300; MT820268; MT820269; MT820279; MT820289; MT820290; MT820291; MT820292; MT820293; MT820294) confirmed the presence of these genes that confer resistance to β-lactam and fluoroquinolone antimicrobials.

**Table 2:** Prevalence of β-lactamase-, and PMQR-encoding genes in *E. coli* and *Salmonella* spp. isolated from cloacal swab and sewage samples.

Resistance genes	No. (%) of isolates			
	Cloacal swab		Sewage samples	
	*E. coli* (n = 44)	*Salmonella* spp. (n = 38)	*E. coli* (n = 5)	*Salmonella* spp. (n = 5)
**BSBL-encoding genes**				
*bla*TEM	44 (100.0)	38 (100.0)	5 (100.0)	5 (100.0)
*bla*SHV	0	0	1 (20.0)	0
**ESBL-encoding genes**				
*bla*CTX-M-1	0	0	0	0
*bla*CTX-M-2	0	0	0	0
**PMQR-encoding genes**				
*qnr*A	4 (9.1)	0	0	0
*qnr*B	0	0	0	0
*qnr*S	31 (70.5)	1 (2.6)	4 (80.0)	0

*n* No. of isolates, *BSBL* broad-spectrum β-lactamase, *ESBL* extended-spectrum β-lactamase, *PMQR* plasmid-mediated quinolone resistance.

### Prevalence of *mecA* gene in *S. aureus*

In case of *S. aureus*, the presence of *mecA* gene was detected in 47.2% (17/36) of cloacal swab and 25% (1/4) of sewage samples.

### Coincidence of resistance genes

Among *E. coli* isolates, the most frequent gene combinations were a two-gene pattern of *bla*TEM + *qnrS* in cloacal swab (n = 31) and sewage samples (n = 3), followed by *bla*TEM + *qnrA* (n = 4) in cloacal swab samples. However, one isolate of sewage samples carried three genes combination (*bla*TEM + *bla*SHV + *qnrS*). Furthermore, coincidence of *bla*TEM with *qnrS* was also identified as the most prevalent combination in case of *Salmonella* spp. recovered from cloacal swab samples. However, no coincidence of resistance genes in *Salmonella* spp. was found in case of sewage samples (Table 3).

**Table 3:** Coincidence of resistance genes among *E. coli* and *Salmonella* spp. isolated from cloacal swab and sewage samples.

Bacteria	Source	Patterns of resistance genes	No. of isolates
*E. coli*	Cloacal swab	*bla*TEM	9
		*bla*TEM + *qnrA*	4
		*bla*TEM + *qnrS*	31
		Total	44
	Sewage samples	*bla*TEM	1
		*bla*TEM + *qnrS*	3
		*bla*TEM + *bla*SHV + *qnrS*	1
		Total	5
*Salmonella* spp.	Cloacal swab	*bla*TEM	37
		*bla*TEM + *qnrS*	1
		Total	38
	Sewage samples	*bla*TEM	5

## Discussion

The pilot sink survey reports the baseline findings on the extent and distribution of foodborne bacteria such as *E. coli*, *Salmonella* spp., and *S. aureus* alongside their antimicrobial resistance pattern including resistance genes in cloacal swab and sewage samples from five wholesale chicken markets in Dhaka city of Bangladesh. This investigation indicated the high occurrence of *E. coli* (88% vs 100%), *Salmonella* spp. (76% vs 100%), and *S. aureus* (72% vs 80%) in cloacal swab and sewage samples, respectively. These findings are in accordance with previous studies in Bangladesh where similar occurrences of *E. coli* (83% vs 85%), *Salmonella* spp. (71% vs 95%), and *S. aureus* (87% vs none) were reported in cloacal swab and sewage samples, respectively^21,26,30,31^. The variable occurrence of *E. coli* (9.2–84%) in cloacal swab samples was reported earlier in Egypt (9.2%)^20^, Japan (84%)^32^, Portugal (42.1%)^19^, and Belgium (60.3%)^33^. Besides, the predominance of *Salmonella* spp. isolated from cloacal swab samples differed from 10.2% in South Korea^34^, 26.8% in India^35^, and 32% in Egypt^8^, and *S. aureus* varied from 10% in India^36^, 19.1% in Indonesia^37^, and 84.8% in Iraq^15^. In the current study, mixed isolation of *E. coli*, *Salmonella* spp. and *S. aureus* were observed in 90% of cloacal swab and 100% of sewage samples. These three foodborne bacteria are usually present in the gut of chickens, and faecal shedding allows these bacteria to be transmitted among chickens, and in the environment of the wholesale markets^7^.

ESBL-producing microorganisms are far reaching around the world including Bangladesh^10,19^. Accordingly, antimicrobial resistance inferable from ESBLs is a significant general wellbeing concern. In the current investigation, the prevalence of ESBL-producing *E. coli*, and *Salmonella* spp. from cloacal swab samples was 98%, and 76%, individually. The rate of ESBL-positive *E. coli* and *Salmonella* spp. was higher than those found in feces of chickens, 30% ESBL-producing *E. coli* in Bangladesh^9^, and 13.1% ESBL-producing *Salmonella* spp. in Belgium^38^. Similarly, the isolation rate of ESBL-producing *E. coli*, and *Salmonella* spp. was also higher in sewage samples (100%, and 60%, respectively). Prevalence of 17.2–29.5% ESBL-producing *E. coli* from water samples has been reported previously in Bangladesh^10,24,25^. The high prevalence of ESBL-producing *E. coli* and *Salmonella* spp. in cloacal swab and sewage samples might be due to widespread use of broad-spectrum antimicrobials in poultry production, ESBL–producing bacteria have evolved and shown greater incidence owing to mutations, selection, and the spread of ARGs in chickens and environment^39^.

Methicillin-resistant *S. aureus* (MRSA), which is present in chickens and sewage samples may serve as a reservoir for MRSA, accordingly permitting this microorganism to continue and spread in the community. The consequences of this investigation demonstrated a high prevalence (47% vs 50%) of MRSA in cloacal swab and sewage samples, separately. This is inconsistent with the result of previous reports, in which 19.2% prevalence of MRSA was observed in cloacal swab from broiler chickens in Chattogram district of Bangladesh^40^. Prevalence of MRSA in cloacal swab and environmental samples were 27.3%, and 8.3% in Iraq and Netherlands, respectively^15,16^. The variation observed in this study might be attributed to differences in the regulation of antibiotics used during poultry production systems and hygiene management of wholesale markets^41^.

An important finding of concern in this study is that 93% *E. coli*, 100% *Salmonella* spp., and 97% *S. aureus* isolates from cloacal swab samples were MDR. Results similar to ours were reported from Bangladesh, Egypt, and India where MDR *E. coli* isolates from cloacal swab samples were 100%, 98.2%, and 94%, respectively^18,20,42^, and MDR *Salmonella* spp. ranged from 91.5–100% in China^43,44^. Furthermore, a higher number of MDR *E. coli*, *Salmonella* spp., and *S. aureus* was also found from sewage samples. But findings contrasting to ours were reported from Bangladesh, India, and South Korea^25,34,45^. A significant proportion of the isolates among these three types of bacteria from both samples were resistant to 6–8 antimicrobial classes. A high occurrence of MDR *E. coli*, *Salmonella* spp., and *S. aureus* isolated from both samples was also observed in different markets. Moreover, the emergence of extensively drug resistance (XDR) in foodborne bacteria has become a noteworthy public health threat because of very few, or even sometimes no, antibiotics can be effective for infections caused by these bacteria. Prominently, the current examination additionally saw that 8.3% of *S. aureus* isolates from cloacal swab samples were possible extensively drug resistant (pXDR). A previous report from India identified XDR *S. aureus* in 15.1% of clinical samples in humans^46^. The reason behind the high MDR and existence of pXDR among these foodborne bacteria in this study might be attributed to the selective pressure because of unnecessary use of antimicrobials as feed added substances or prophylactic medicines in chickens and the environment where chickens are raised. Because of abuse of antimicrobials, microorganisms with MDR may at last supplant drug-touchy microorganisms in conditions soaked with antimicrobial agents^47^. In Bangladesh, antimicrobials are easily accessible, and can be bought without prescription by the veterinarians. In this way, exacting rules and comprehensive antimicrobial medication monitoring frameworks should be earnestly advocated and implemented particularly in developing countries like Bangladesh for the rational use of antimicrobial agents in poultry production to diminish the development of MDR and pXDR isolates.

Fluoroquinolones are considered as medications of decision for treatment of human infections caused by Gram-negative and Gram-positive microbes^48^. However, the development of resistance to fluoroquinolones has been arisen throughout the time because of abuse and additionally overdose of medications in human and veterinary practice thus of expanding general wellbeing concern^49^. Nalidixic acid, the first generation quinolone, has the ability to develop resistance quite rapidly. In this current investigation, nalidixic acid resistance was the most as often as possible noticed antimicrobial resistance in *E. coli*, *Salmonella* spp., and *S. aureus* from cloacal swab and sewage samples. Moreover, resistance to pefloxacin was found higher among *E. coli* and *Salmonella* spp. in both samples in this study. A few studies have likewise indicated that resistance to nalidixic acid and decreased susceptibility to fluoroquinolones have expanded among *E. coli*, *Salmonella* spp., and *S. aureus* from chickens and environment^10,23,26,34,37,50^.

A high percentages of *E. coli*, *Salmonella* spp., and *S. aureus* recovered from cloacal swab and sewage samples were resistant to ampicillin, trimethoprim-sulfamethoxazole, and doxycycline, which is as per past reports conducted in Bangladesh^18,23,50,51^, Egypt^8^, and India^36^. The high resistance to ampicillin, trimethoprim-sulfamethoxazole, and doxycycline indicates that these antimicrobials have been persistently utilized in enormous amounts in the poultry production in Bangladesh. These perceptions uphold the likelihood that chickens might be a possible source of antimicrobial-resistant foodborne infections in humans^52^.

Of note, about 60% of *E. coli* isolates were resistant to imipenem and meropenem, and 40% of *Salmonella* spp. to imipenem in cloacal swab and sewage samples though this two antimicrobials are not used in poultry practices in Bangladesh. Our findings expressed a higher percentage of carbapenem resistance of *E. coli* and *Salmonella* spp. contrasted with previous investigation on chickens and environmental samples, were found to be 1.8% in Egypt^20^, 2.9% in Philippines^53^, and 3.1–8.1% in India^45,54^. Development of carbapenem resistance in *E. coli* and *Salmonella* spp. may be because of horizontal gene exchange of carbapenemase-encoding genes together with co- and cross-selective mechanisms^55^. If once acquired carbapenem resistance, at that point this resistance can be transmitted from humans to poultry and from poultry to humans through the food chain. Global epidemiological observation of resistance to these “last resort” antimicrobials is needed to build up potential connections between reservoirs and to restrict the bidirectional exchange of the encoding genes between foodborne bacteria and other commensal bacteria^55^.

Colistin as antimicrobial substance has been widely used in poultry production in many countries including Bangladesh, especially for prevention and treatment of *Enterobacteriaceae* infections and as growth promotion purposes^56^. According to WHO, colistin has been reclassified as an antibiotic of highest priority critically importance to treat infections caused by MDR and carbapenem-resistant bacteria in human medicine^57^. The extensive use of colistin in humans and poultry is recognized as the reason for the emergence and dissemination of the colistin resistance. In the present study, almost all the isolates of *Salmonella* spp., and 50% isolates of *S. aureus* obtained from cloacal swab and sewage samples were resistant to colistin. It is much higher compared to the findings of Aditya^58^ who reported 50% of the *Salmonella* spp. isolates of chickens were resistant to colistin. But, another study reported 75% of the *S. aureus* isolates from environmental samples showed resistant to this antimicrobial^50^, which is higher than our study. The long term prophylactic use of colistin in poultry production may be attributed to this high resistance rate, which can be spread by direct or indirect poultry-to-human contact.

In this investigation, it was noticed that the *S. aureus* isolates from cloacal swab and sewage samples had the highest antibiotic resistance to ceftazidime (100%), oxacillin (89%), and cefixime and cloxacillin (75–81%). The oxacillin-resistant *S. aureus* was previously isolated from 32.1% of chicken samples^15^, which was in conflict with our outcomes. The purpose behind the distinction in resistance rates may be a fast change in antimicrobial sensitivity patterns of bacteria inside a brief period^5^.

Among the predominant β-lactamase genes responsible for β-lactam antibiotic resistance, *bla*TEM, *bla*SHV, and *bla*CTX-M (*bla*CTX-M-1 and *bla*CTX-M-2) are viewed as generally assorted. The β-lactamase genes are generally located on plasmids, which could advance the dissemination of β-lactamase genes in Gram-negative bacteria^13^. The *bla*TEM gene was the most prevalent broad-spectrum β-lactamase (BSBL)-encoding gene found in all the isolates of *E. coli* and *Salmonella* spp. from cloacal swab and sewage samples, which is consistent with previous studies conducted in Bangladesh^18^, Portugal^19^, and China^43^. Another BSBL-encoding gene, the *bla*SHV, was detected in one *E. coli* isolate from cloacal swab samples. Very little data is accessible on the event of β-lactamase encoding genes in isolates from chickens in Bangladesh, yet comparative outcomes have been accounted for in studies somewhere else^20,53^. No *bla*CTX-M-1 and *bla*CTX-M-2 ESBL-encoding genes were detected in any isolates of *E. coli* and *Salmonella* spp. in this study. These findings are conflicting with prior investigations in Bangladesh, where over 90% of *E. coli* isolates from droppings of chickens and water samples harboured the *bla*CTX-M-1 gene^9,24^. Presence of β-lactamase genes on genetic mobile elements can encourage their exchange across bacterial species or genera^13^.

Plasmid-mediated quinolone resistance (PMQR) spoke to by quinolone resistance (*qnr*) genes is generally distributed among Gram-negative bacteria including Bangladesh^10,20^. PMQR genes have been reported to be carried on portable gene elements, and can be easily moved among various bacterial strains and species. This potential intensifies the development of multidrug resistance in light of the fact that PMQR supposedly decreases microbial susceptibility to antimicrobials and supports the event of resistance-associated mutations on bacterial chromosomes, accordingly making *Enterobacteriaceae* infections significantly more hard to treat^59^. In the current study, *qnrS* gene was detected in 70.5% of *E. coli* and 80% of *Salmonella* spp. isolates from cloacal swab samples. In Bangladesh, *qnrS* gene was detected with similar percentage (72.2%) of *E. coli* isolates acquired from cloacal swab samples in Mymensingh district^26^. However, in Uganda, *qnrS* gene was identified with low percentage (18.8%) of *Salmonella* spp. recovered from chickens^60^. Furthermore, 2.6% of *E. coli* isolates from sewage samples also harboured the *qnrS* gene, which is comparatively lower than the study conducted earlier in Bangladesh^24^. Moreover, *qnrA* gene was detected only in 9.1% of the *E. coli* isolates from cloacal swab samples which is inconsistent with the previous report, who detailed none of the *E. coli* isolates from chickens harboured *qnrA* gene^26^. High presence of the PMQR gene *qnrS* hence shows the capability of horizontal transfer of resistance genes^13^.

In this investigation, the occurrence of *mecA* gene in *S. aureus* isolated from cloacal swab and sewage samples were 47.2% and 25%, individually, which is significantly higher than that reported beforehand in Bangladesh^21^. The high occurrence of *mecA* gene in cloacal swab and sewage samples in our investigation showed that the overuse of antibiotics in poultry production that eliminate methicillin-sensitive *Staphylococcus* and encourage MRSA colonization.

The coexistence of β-lactamase and PMQR genes in *E. coli* and *Salmonella* spp. emerges a genuine worry to people. Notably, thirty one *E. coli* isolates from cloacal swab samples carried *bla*TEM and *qnrS* gene, while four isolates carried *bla*TEM and *qnrA* gene. These outcomes are in concurrence with the findings of prior study in China, which exhibit that the presence of β-lactamase and PMQR genes in the similar *E. coli* strain^22^. Likewise, *bla*TEM, *bla*SHV, *qnrS* genes were coexisted in one *E. coli* isolate from sewage samples. Until now, this is the first time that *bla*TEM, *bla*SHV, *qnrS* have been found to coexist in an individual *E. coli* isolate in Bangladesh. One *Salmonella* spp. isolates from cloacal swab samples possessed a combination of *bla*TEM and *qnrS* gene. Further, our information associate with previously referenced studies concerning the coexistence of various resistance mechanisms in one isolate^61^. β-Lactamase genes are often harboured on plasmids containing other resistance genes, e.g., PMQR gene, accordingly, the use of β-lactam antimicrobials enables their co-selection^61^. Further molecular analyses could be performed to build up the relatedness of the foodborne bacteria from the chicken samples to human isolates since the antimicrobial resistance genes assessed in this study can be easily moved to poultry and human strains. Furthermore, further study on the isolates should be led to depict the association between the presence and level of expression of the selected genes. To prevent the spread of antimicrobial resistance, further research using large sample collections is needed to better understand the molecular genetic pathways involved in the spread of antimicrobial resistance genes from foodborne pathogens to people. The results of the occurrence of foodborne bacteria and their pattern of antimicrobial resistance should be utilized with caution because few samples were collected and examined at a time point though the samples were representative of the target population. To extrapolate the results of our pilot study to the broader population, a follow-up study with large sample size is warranted.

In conclusion, our baseline data indicate the presence of common foodborne MDR bacteria in cloacal swab of broiler chickens sold in the wholesale chicken markets of Dhaka city of Bangladesh and the respective sewage samples, along with the existence of β-lactamase, plasmid mediated quinolone resistance, and methicillin resistance genes. The findings envisage the potential public health risk and environmental health hazard through spillover of common foodborne MDR bacteria. The results highlight the need for rapid implementation of an integrated program for surveillance of antimicrobial resistance in order to monitor trends, raise awareness, and improve practices with special emphasis to sanitary sewage system to safeguard next generation antimicrobial agents.

## Materials and methods

### Study design and areas

**Figure 5:**
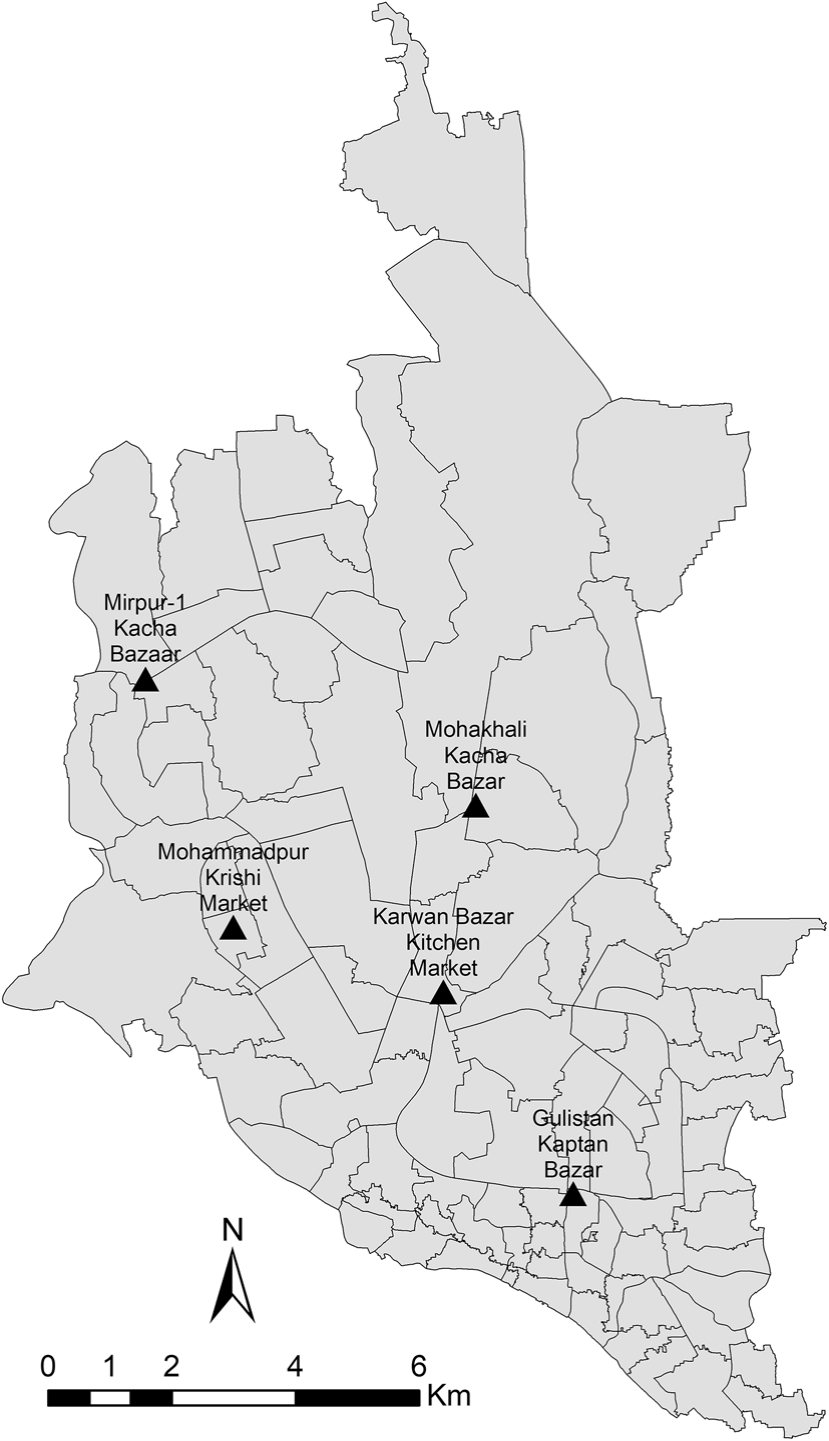
Map showing five wholesale chicken markets (▲) in Dhaka city of Bangladesh. [The map was generated by using ArcGIS 10.4.1 for Desktop software, https://desktop.arcgis.com/en/arcmap/10.4/].

A cross-sectional sink survey was conducted in five wholesale chicken markets (Karwan Bazar Kitchen Market, Mohakhali Kacha Bazar, Gulistan Kaptan Bazar, Mohammadpur Krishi Market, and Mirpur-1 Kacha Bazar) in Dhaka city of Bangladesh during November to December 2019 (Fig. 5). The wholesale chicken markets of Dhaka city are the hubs where the chickens are brought from different parts of Bangladesh, and these chickens are distributed to different market places of the city.

### Sample and data collection

A total of 50 pooled cloacal swab samples from broiler chickens (10 from each market) were collected very early in the morning from five wholesale chicken markets. Sampling was done from 10 wholesale poultry stalls per market and 10 swab samples were collected from each stall and then pooled. In addition, five pooled sewage samples from dedicated sewage systems for the wholesale market, having one sample (pooled from five individual samples) from each market were collected. Data on origin of chickens, type of chickens sold, market distribution, daily average sales size, waste disposal, and drainage facilities were also collected.

### Sample processing

Cloacal swab samples were collected by using sterile swab sticks. The exterior of the cloaca of chickens was cleaned with cotton soaked with 70% alcohol, and a sterile swab stick was inserted into the cloaca. Then the swab stick was dipped directly into the sterile collection tube containing 1 mL of sterile buffered peptone water (BPW). Besides, sewage water sample (~ 150 mL) was taken aseptically in a sterile falcon tube. After collection, all samples were kept in a cool box with ice packs, and transported to the laboratory on the same day. On getting to the laboratory, bacteria in sewage water were pelleted by centrifugation at 600×*g* for 20 min, and re-suspended in 1 mL of BPW. For the pre-enrichment of bacteria, both types of samples with BPW were incubated at 37 °C for 24 h. From that point onward, each sample was transferred into two separate test tubes; one containing the nutrient broth (NB) for isolation of *E. coli* and *S. aureus*, and another one containing Rappaport-Vassiliadis Soya broth (RVS) for isolation of *Salmonella* spp., and incubated for 24 h at 37 °C for selective enrichment^62^.

### Isolation and identification of common foodborne bacteria

After selective enrichment, a loopful of the NB culture was concurrently streaked onto Eosin Methylene Blue (EMB) agar and Mannitol Salt Agar (MSA) in duplicate for the isolation of *E. coli* and *S. aureus*, respectively, while a loopful of the RVS culture was streaked onto Xylose-Lysine-Deoxycholate (XLD) agar in duplicate for the isolation of *Salmonella* spp., and incubated at 37 °C for 18–24 h. Distinct colonies having a dark blue colour with a characteristic metallic sheen on EMB, a black center and a slightly transparent zone of reddish colour on XLD, and yellow colour colonies with yellow zones on MSA were presumptive for *E. coli*, *Salmonella* spp., and *S. aureus*, respectively. Three presumptive colonies from each selective agar plate were picked, and then subcultured to obtain a pure culture. Gram staining and biochemical tests such as catalase, oxidase, indole, methyl red, Voges–Proskauer tests, a sugar fermentation test using triple sugar iron agar for *E. coli* and *Salmonella* spp., and catalase and coagulase tests for *S. aureus* were performed from the pure culture. Positive isolates were stored in nutrient broth containing 50% (v/v) buffered glycerol at − 20 °C for further study.

### Molecular detection of common foodborne bacteria

Biochemically positive isolates of the bacteria were confirmed by polymerase chain reaction (PCR) assay. For molecular detection, the pure isolates of the organisms were sub-cultured overnight in NB and genomic DNA was extracted by using the “boiling” method as described by Dashti et al.^63^. Two uniplex PCR targeting *malB* promoter gene and *ITS* gene were used for the confirmation of *E. coli* and *Salmonella* genus, respectively. On the other hand, a duplex PCR was carried out for the confirmation of *S. aureus* with two sets of genus- and species-specific primers. Primers used for *E. coli*: ECO-1 (5′-GACCTCGGTTTAGTTCACAGA-3′) and ECO-2 (5′-CACACGCTGACGCTGACCA-3′) for the amplification of 585 bp^64^; for *Salmonella* spp.: ITSF (5′-TATAGCCCCATCGTGTAGTCAGAAC-3′) and ITSR (5′-TGCGGCTGGATCACCTCCTT-3′) for the amplification of 312 bp^65^; and for *S. aureus*: Staph756F (5′-AACTCTGTTATTAGGGAAGAACA-3′) and Staph750R (5′-CCACCTTCCTCCGGTTTGTCACC-3′ for the amplification of 756 bp from *16S rRNA* gene^66^, and Nuc450-F (5′-AGTATATAGTGCAACTTCAACTAAA-3′) and Nuc450-R (5′-ATCAGCGTTGTCTTCGCTCCAAATA-5′) for the amplification of 450 bp from *nuc* (thermonuclease) gene^67^. Amplification reactions and PCR conditions are described in Supplementary Table S5. After amplification, PCR products were analysed by gel electrophoresis on 1.5% UltraPure™ Agarose gel stained with ethidium bromide (5 µg/mL) including a 100-bp DNA ladder (BioLabs, New England) which served as a molecular weight marker. The resulting band of PCR product was visualized under UV transilluminator and photographed.

### Antimicrobial susceptibility testing

The AMR profile of all *E. coli*, *Salmonella* spp., and *S. aureus* isolates were determined using the Kirby-Bauer disk diffusion method as described by the Clinical and Laboratory Standards Institute (CLSI)^68^. A panel of 31 antimicrobials representing 15 different antimicrobial classes was used for *E. coli* and *Salmonella* spp., and 27 antimicrobials belonging to 11 antimicrobial classes were used for *S. aureus*. The antimicrobials commonly used for antimicrobial susceptibility testing include, the fluoroquinolones [nalidixic acid (NA, 30 µg), ciprofloxacin (CIP, 5 µg), levofloxacin (LEV, 5 µg), norfloxacin (NX, 10 µg), ofloxacin (OFX, 5 µg), gatifloxacin (GAT, 5 µg), pefloxacin (PEF, 5 µg)], non-extended spectrum cephalosporins [first-generation cephalosporins: cephalexin (CL, 30 µg), cephradine (CE, 30 µg); second-generation cephalosporins: cefuroxime (CXM, 30 µg), cefaclor (CEC, 30 µg)], extended-spectrum cephalosporins [third-generation cephalosporins: cefotaxime (CTX, 30 µg), ceftriaxone (CRO, 30 µg), ceftazidime (CAZ, 30 µg), cefixime (CFM, 5 µg); fourth-generation cephalosporins: cefepime (FEP, 30 µg)], cephamycins [cefoxitin (FOX, 30 µg)], carbapenems [imipenem (IPM, 10 µg), meropenem (MEM, 10 µg)], tetracyclines [doxycycline (DO, 10 µg)], penicillins [ampicillin (AM, 10 µg), methicillin (MET, 5 µg), oxacillin (OX, 1 µg), cloxacillin (CX, 5 µg)], penicillins + β-lactamase inhibitors [amoxicillin-clavulanic acid (AMC, 30 µg)], antipseudomonal penicillins + β-lactamase inhibitors [piperacillin–tazobactam (TPZ, 110 µg)], aminoglycosides [gentamicin (CN, 10 µg), amikacin (AK, 30 µg)], monobactams [aztreonam (AT, 30 µg)], folate pathway inhibitors [trimethoprim-sulfamethoxazole (SXT, 25 µg), glycylcyclines [tigecycline (TGC, 15 µg)], phenicols [chloramphenicol (C, 30 µg)], glycopeptides and lipoglycopeptides [vancomycin (VA, 30 µg)], polymyxins [colistin (CT), polymyxin B (PB)]. However, ampicillin, piperacillin–tazobactam, amikacin, aztreonam, trimethoprim-sulfamethoxazole, tigecycline, chloramphenicol and polymyxin B used for *E. coli* and *Salmonella* spp., and methicillin, oxacillin, cloxacillin, and vancomycin used for *S. aureus* only. For colistin, polymyxin B and vancomycin, minimum inhibitory concentrations (MICs) were determined by broth microdilution method.

The interpretive category (susceptible, intermediate, and resistant) of each isolate was determined according to the CLSI guidelines^68^, and in some cases when breakpoints of some antimicrobials in CLSI were unavailable, the guideline of European Committee on Antimicrobial Susceptibility Testing (EUCAST) was used^69^. Extended spectrum β-lactamases (ESBL)-producing *E. coli* and *Salmonella* spp., and methicillin-resistant *S. aureus* (MRSA) were detected by using the double-disk synergy method and cefoxitin disk diffusion method, respectively^68^.

### Definition of MDR and pXDR

Isolates resistant to at least one agent in three or more antimicrobial classes were defined as multidrug resistance (MDR) while isolates resistant to at least one agent in all but two or fewer antimicrobial classes i.e. bacterial isolates remain susceptible to three classes were defined as possible extensively drug resistant (pXDR)^70^.

### Detection of antimicrobial resistance genes

#### Detection of β-lactamase and plasmid-mediated quinolone resistance (PMQR) genes

The presence of β-lactamase-encoding genes (broad-spectrum β-lactamases: *bla*TEM, *bla*SHV, and extended spectrum β-lactamases: *bla*CTX-M-1, *bl*aCTX-M-2), and PMQR genes (*qnrA*, *qnrB*, and *qnrS*) in *E. coli* and *Salmonella* spp. were determined by two separate multiplex PCR with specific primers listed in Supplementary Table S6. Details of the PCR protocol and thermal profile used for detection of β-lactamase, and PMQR genes were described in our previous study^71^. A representative numbers (*bla*TEM—8, *bla*SHV—1, *qnrA*—2, and *qnrS*—7) of PCR products were sequenced from commercial service provider (Macrogen Inc., Seoul, Korea). The identification of sequences was confirmed by comparison with known sequences in GenBank by using the BLAST program (National Center for Biotechnology Information, USA).

#### Detection of methicillin resistance gene

A uniplex PCR targeting methicillin resistance gene (*mecA*) in *S. aureus* was standardized, and used in this study with specific primer listed in Supplementary Table S6^66^. Each PCR reaction mixture was constituted in a final reaction mixture of 25 µL made up of 12.5 µL PCR master mix (Thermo Fisher Scientific, Waltham, MA, USA), 1.5 µL (15 pmol) each of forward and reverse primers, 7.5 µL of nuclease-free water, and 2 µL of DNA template. Amplification was performed with this thermal profile: heating at 94 °C for 5 min, followed by 30 cycles of denaturation at 94 °C for 1 min, primer annealing at 55 °C for 1 min, extension at 72 °C for 2 min, and a final extension step for 10 min at 72 °C.

### Data analyses

Data were entered into spread sheet (Microsoft Excel 2010) and transferred into SPSS software v22.0 (IBM Corp., Armonk, NY, USA) for statistical analysis. Descriptive statistics were used to compute the prevalence of bacteria and resistance percentage. The significant differences in prevalence of bacteria and resistance percentage among sample types, sampling area were determined using Chi-square test (*Z*-test for proportions) and Fisher's exact test (wherever appropriate). The level of significance was set at p < 0.05. An UpSet plot was constructed to show the antimicrobial resistance pattern of *E. coli*, *Salmonella* spp. and *S. aureus*, and a Venn diagram to find out the co-isolation of bacteria was drawn by using online tools^72,73^. Map was generated to show the sampling sites in Dhaka city of Bangladesh using ArcGIS 10.4.1 Software (ESRI Redlands, NY, USA).

### Ethical consideration

This study was approved by the Animal Welfare and Experimentation Ethics Committee of Bangladesh Agricultural University, Mymensingh. The approval number was AWEEC/BAU/2017(13). No animal experimentation was done in this study. However, informed written consent was taken from the live bird shop owners before sampling from broiler chickens. Cloacal swab samples were collected from the broiler chickens causing minimal distress to the chickens as per OIE guidelines^74^.

## Supplementary Information

Supplementary Information.

## Data Availability

The datasets generated and analyzed during the current study are available from the corresponding author on reasonable request.
