# Supplementary material for: Sink survey to investigate multidrug resistance pattern of common foodborne bacteria from wholesale chicken markets in Dhaka city of Bangladesh
Source: Sci Rep. 2022 Jun 25;12:10818. doi: 10.1038/s41598-022-14883-7 (PMC9233690; doi:10.1038/s41598-022-14883-7)

**Supplementary Table S1.** Demographic information of five wholesale chicken markets in Dhaka city.

| **Name of Markets** | **Karwan Bazar Kitchen Market** | **Gulistan Kaptan Bazar** | **Mirpur-1 Kacha Bazar** | **Mohakhali Kacha Bazar** | **Mohammadpur Krishi Market** |
| --- | --- | --- | --- | --- | --- |
| **Daily average sales size** | | | | | |
| 5000-10000 |  |  |  |  |  |
| >10000 |  |  |  |  |  |
| **Type of birds sold** | | | | | |
| Broiler |  |  |  |  |  |
| Layer |  |  |  |  |  |
| Cockerel |  |  |  |  |  |
| Backyard chickens |  |  |  |  |  |
| Turkey |  |  |  |  |  |
| Duck |  |  |  |  |  |
| Pigeon |  |  |  |  |  |
| **Market distribution** (retail markets, supershops, hotel, restaurant, community center) |  |  |  |  |  |
| **Waste disposal facility** |  |  |  |  |  |
| **Waste water disposal facility** |  |  |  |  |  |
| **Drainage facility** |  |  |  |  |  |

Present

Absent

**Supplementary Table S2.** Market-wise prevalence of *E. coli*, *Salmonella* spp. and *S. aureus* isolated from cloacal swab and sewage samples**.**

| **Name of wholesale chicken market** | **Cloacal swab** | | | | **Sewage samples** | | | |
| --- | --- | --- | --- | --- | --- | --- | --- | --- |
|  | **No. of samples** | ***E. coli***  **(N, %)** | ***Salmonella* spp. (N, %)** | ***S. aureus***  **(N, %)** | **No. of samples** | ***E. coli***  **(N, %)** | ***Salmonella* spp. (N, %)** | ***S. aureus***  **(N, %)** |
| Karwan Bazar Kitchen Market | 10 | 10 (100)^a^ | 7  (70)^ab^ | 7  (70)^a^ | 1 | 1  (100)^a^ | 1  (100)^a^ | 1  (100)^a^ |
| Gulistan Kaptan Bazar | 10 | 9  (90)^ac^ | 9  (90)^a^ | 7  (70)^a^ | 1 | 1  (100)^a^ | 1  (100)^a^ | 1  (100)^a^ |
| Mirpur-1 Kacha Bazar | 10 | 6  (60)^b^ | 8  (80)^ab^ | 7  (70)^a^ | 1 | 1  (100)^a^ | 1  (100)^a^ | 1  (100)^a^ |
| Mohakhali Kacha Bazar | 10 | 10 (100)^a^ | 6  (60)^b^ | 7  (70)^a^ | 1 | 1  (100)^a^ | 1  (100)^a^ | 0 |
| Mohammadpur Krishi Market | 10 | 9  (90)^ac^ | 8  (80)^ab^ | 8  (80)^a^ | 1 | 1  (100)^a^ | 1  (100)^a^ | 1  (100)^a^ |
| Total | 50 | 44 (88.0) | 38  (76.0) | 36  (73.0) | 5 | 5 (100.0) | 5  (100.0) | 4  (80.0) |

N = No. of isolates, ^a,b,c^Values in the same column with different letters differ significantly (p ≤ 0.05).

**Supplementary Table S3.** Wholesale chicken market-wise distribution of MDR *E. coli*, *Salmonella* spp. and *S. aureus* isolated from cloacal swab and sewage samples.

| **Name of wholesale chicken market** | **Cloacal swab** | | | **Sewage samples** | | |
| --- | --- | --- | --- | --- | --- | --- |
|  | ***E. coli***  **(N, %)** | ***Salmonella* spp. (N, %)** | ***S. aureus***  **(N, %)** | ***E. coli***  **(N, %)** | ***Salmonella* spp. (N, %)** | ***S. aureus***  **(N, %)** |
| Karwan Bazar Kitchen Market | 9 (90)^a^ | 7 (100)^a^ | 7 (100)^a^ | 1 (100)^a^ | 1 (100)^a^ | 1 (100) |
| Gulistan Kaptan Bazar | 9 (100)^a^ | 9 (100)^a^ | 7 (100)^a^ | 0 | 1 (100)^a^ | 1 (100) |
| Mirpur-1 Kacha Bazar | 6 (100)^a^ | 8 (100)^a^ | 7 (100)^a^ | 1 (100)^a^ | 1 (100)^a^ | 1 (100) |
| Mohakhali Kacha Bazar | 10 (100)^a^ | 6 (100)^a^ | 6 (85.7)^a^ | 1 (100)^a^ | 1 (100)^a^ | 1 (100) |
| Mohammadpur Krishi Market | 7 (77.8)^a^ | 8 (100)^a^ | 8 (100)^a^ | 1 (100)^a^ | 1 (100)^a^ | 1 (100) |
| **Total** | **41 (93.2)** | **38**  **(100)** | **35**  **(97.2)** | **4 (80.0)** | **1**  **(100)** | **1**  **(100)** |

Values in the same column with same superscript do not differ significantly (p ≥ 0.05).

**Supplementary Table S4.** Individual antimicrobial resistance pattern of *E. coli*, *Salmonella* spp. and *S. aureus* isolated from sewage samples.

| **Antimicrobial agents** | **No. (%) of resistant isolates** | | |
| --- | --- | --- | --- |
|  | ***E. coli***  **(n = 5)** | ***Salmonella* spp.**  **(n = 5)** | ***S. aureus***  **(n = 4)** |
| **Fluoroquinolones** | | | |
| Ciprofloxacin | 2 (40.0) | 0 | 2 (50.0) |
| Nalidixic acid | 3 (60.0) | 5 (100) | 3 (75.0) |
| Levofloxacin | 2 (40.0) | 1 (20.0) | 2 (50.0) |
| Norfloxacin | 1 (20.0) | 1 (20.0) | 1 (25.0) |
| Gatifloxacin | 2 (40.0) | 2 (40.0) | 2 (50.0) |
| Pefloxacin | 5 (100) | 5 (100) | 2 (50.0) |
| Ofloxacin | 2 (40.0) | 2 (40.0) | 3 (75.0) |
| **Non-extended spectrum cephalosporins** | | | |
| **1^st^ generation** | | | |
| Cephalexin | 1 (20.0) | 1 (20.0) | 1 (25.0) |
| Cephradine | 0 | 0 | 0 |
| **2^nd^ generation** | | | |
| Cefuroxime | 0 | 0 | 1 (25.0) |
| Cefaclor | 0 | 0 | 0 |
| **Extended-spectrum cephalosporins** | | | |
| **3^rd^ generation** | | | |
| Cefixime | 0 | 0 | 3 (75.0) |
| Ceftazidime | 0 | 0 | 4 (100) |
| Ceftriaxone | 0 | 1 (20.0) | 0 |
| Cefotaxime | 1 (20.0) | 0 | 0 |
| **4^th^ generation** | | | |
| Cefepime | 0 | 0 | 1 (25.0) |
| **Cephamycins** | | | |
| Cefoxitin | 0 | 0 | 2 (50.0) |
| **Penicillins** | | | |
| Ampicillin | 4 (80.0) | 4 (80.0) | - |
| Oxacillin | - | - | 2 (50.0) |
| Cloxacillin | - | - | 3 (75.0) |
| Methicillin | - | - | 0 |
| **Penicillins + *β*-lactamase inhibitors** | | | |
| Amoxicillin-clavulanic acid | 1 (20.0) | 2 (40.0) | 2 (50.0) |
| **Antipseudomonal penicillins + *β*-lactamase inhibitors** | | | |
| Pipercillin-tazobactam | 0 | 0 | - |
| **Carbapenems** | | | |
| Imipenem | 3 (60.0) | 2 (40.0) | 0 |
| Meropenem | 3 (60.0) | 1 (20.0) | 0 |
| **Polymyxins** | | | |
| Colistin | 0 | 5 (100) | 2 (50.0) |
| Polymyxin B | 0 | 5 (100) | - |
| **Monobactams** | | | |
| Aztreonam | 1 (20.0) | 0 | - |
| **Aminoglycosides** | | | |
| Gentamicin | 0 | 3 (60.0) | 1 (25.0) |
| Amikacin | 1 (20.0) | 0 | - |
| **Tetracyclines** | | | |
| Doxycycline | 3 (60.0) | 5 (100) | 3 (75.0) |
| **Folate pathway inhibitors** | | | |
| Trimethoprim-sulfamethoxazole | 5 (100) | 4 (80.0) | - |
| **Glycylcyclines** | | | |
| Tigecycline | 0 | 0 | - |
| **Phenicols** | | | |
| Chloramphenicol | 1 (20.0) | 2 (40.0) | - |
| **Glycopeptides and lipoglycopeptides** | | | |
| Vancomycin | - | - | 0 |

n, number of isolates tested; -, not used

**Supplementary Table S5.** PCR reaction mixture and thermal profile used for detection of *E. coli*, *Salmonella* spp. and *S. aureus*

30

cycles

| **Parameters** | ***E. coli***  (*malB* promoter gene) | ***Salmonella* spp.**  (*ITS* gene) | ***Staphylococcus* spp. and *S. aureus***  (*16S rRNA* and *nuc* genes) |
| --- | --- | --- | --- |
| **Reaction mixture**   - Master mixture^*^ - Primers   - Forward   - Reverse - DNA template - Nuclease-free water | 12.5 μL  1.5 μL (15 pmol)  1.5 μL (15 pmol)  0.5 μL  9.0 μL | 12.5 μL  1.5 μL (15 pmol)  1.5 μL (15 pmol)  1 μL  8.5 μL | 12.5 μL  1.5 μL (15 pmol)  2  sets  1.5 μL (15 pmol)  2 μL  4.5 μL |
| **Thermal profile**   - Initial denaturation - Denaturation - Annealing - Extension - Final extension | 95 °C for 5 min  94 °C for 1 min  35  cycles  58 °C for 1 min  72 °C for 1 min  72 °C for 7 min | 94 °C for 5 min  94 °C for 30 s  35  cycles  60 °C for 30 s  72 °C for 50 s  72 °C for 10 min | 94 °C for 5 min  94 °C for 1 min  55 °C for 1 min  72 °C for 2 min  72 °C for 10 min |

^*^Master mixture: OneTaq® Quick-Load® 2X PCR Master Mix with standard buffer (New England, BioLabs Inc.).

**Supplementary Table S6.** Oligonucleotide primers used for the detection of β-lactamase (BSBL and ESBL), PMQR, and methicillin resistance genes

| **Genes** | **Primers** | **Sequence 5’→ 3’** | **Size (bp)** | **References** |
| --- | --- | --- | --- | --- |
| **BSBL-encoding genes** | | | | |
| *bla*TEM | TEM-410F  TEM-781R | GGTCGCCGCATACACTATTCTC  TTTATCCGCCTCCATCCAGTC | 372 | Le *et al.* (2015) |
| *bla*SHV | SHV-287F  SHV-517R | CCAGCAGGATCTGGTGGACTAC  CCGGGAAGCGCCTCAT | 231 |  |
| **ESBL-encoding genes** | | |  |  |
| *bla*CTX-M-1 | ctxm1-15F  ctxm1-02R | GAATTAGAGCGGCAGTCGGG  CACAACCCAGGAAGCAGGC | 588 |  |
| *bla*CTX-M-2 | ctxm2-39F  ctxm2-45R | GATGGCGACGCTACCCC  CAAGCCGACCTCCCGAAC | 107 |  |
| **PMQR-encoding genes** | | | | |
| *qnrA* | qnrA-F  qnrA-R | ATTTCTCACGCCAGGATTTG GATCGGCAAAGGTTAGGTCA | 516 | Robicsek *et al.* (2006) |
| *qnrB* | qnrB-F  qnrB-R | GATCGTGAAAGCCAGAAAGG ACGATGCCTGGTAGTTGTCC | 469 |  |
| *qnrS* | qnrS-F  qnrS-R | ACGACATTCGTCAACTGCAA TAAATTGGCACCCTGTAGGC | 417 |  |
| **Methicillin resistance gene** | | | | |
| *mecA* | MecA1  MecA2 | GTAGAAATGACTGAACGTCCGATAA  CCAATTCCACATTGTTTCGGTCTAA | 310 | Zhang *et al.* (2004) |

BSBL = Broad-spectrum β-lactamase; ESBL = Extended-spectrum β-lactamase; PMQR = Plasmid-mediated quinolone resistance.

**Supplementary Figure S1.** An UpSet plot showing individual antimicrobial resistance pattern of *E. coli* isolated from cloacal swab samples.


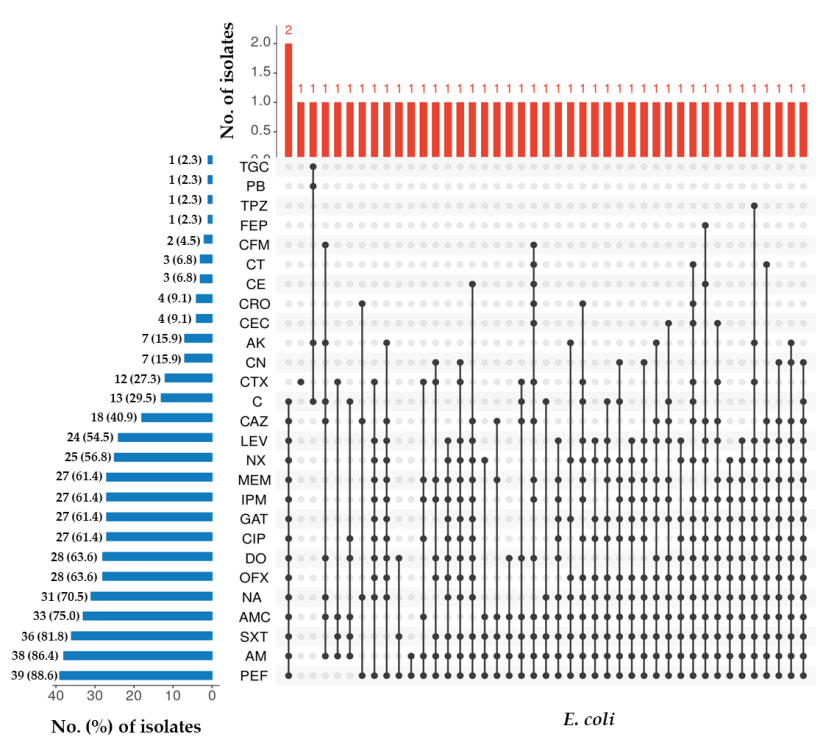


**Supplementary Figure S2.** An UpSet plot showing individual antimicrobial resistance pattern of *Salmonella* spp. isolated from cloacal swab samples.

**
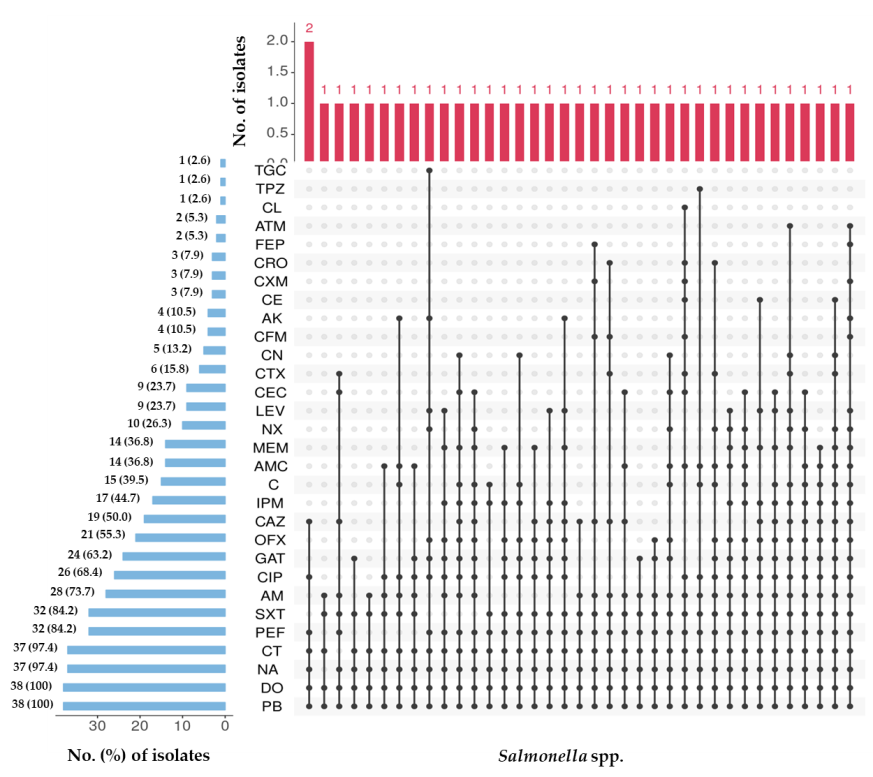
**

**Supplementary Figure S3.** An UpSet plot showing individual antimicrobial resistance pattern of *S. aureus* isolated from cloacal swab samples.


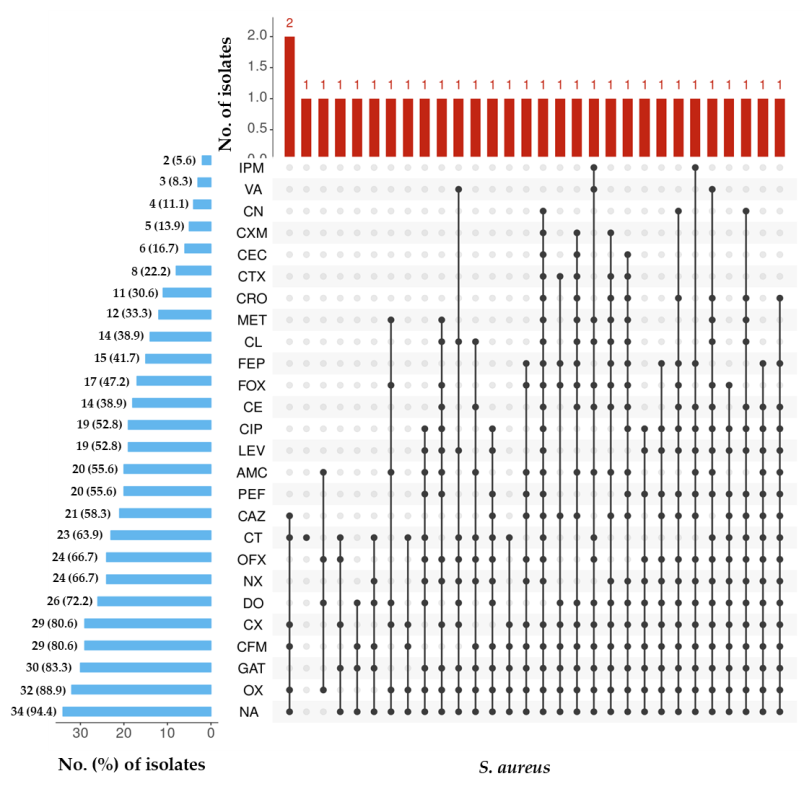

Supplement: Supplementary file 1 — Supplementary Information. [file 41598_2022_14883_MOESM1_ESM.docx]
